# Behavioural biases in the interaction with food objects in virtual reality and its clinical implication for binge eating disorder

**DOI:** 10.1007/s40519-023-01571-2

**Published:** 2023-05-24

**Authors:** Sebastian M. Max, Kathrin Schag, Katrin E. Giel, Christian Plewnia

**Affiliations:** 1 Department of Psychiatry and Psychotherapy, Tübingen Center for Mental Health, University Hospital Tübingen, Neurophysiology & Interventional Neuropsychiatry, Calwerstraße 14, 72076 Tübingen, Germany; 2 Department of Psychosomatic Medicine and Psychotherapy, University Hospital Tübingen, Osianderstraße 5, 72076 Tübingen, Germany; 3 Centre of Excellence for Eating Disorders Tübingen (KOMET), Tübingen, Germany

**Keywords:** Binge eating disorder, Virtual reality, Cognitive control, Behavioural bias

## Abstract

Cognitive processes play a central role in the development, maintenance and remission in mental disorders, like in Binge Eating Disorder (BED). Insights into cognitive mechanisms reflected by embodied interaction with food and its connections to clinically relevant psychopathology offer new possibilities for translational diagnostics and interventions. We longitudinally investigated the manual interaction with food in a virtual reality (VR) in 31 patients with BED. Patients were assessed at baseline before participating in a randomized-controlled trial (RCT) investigating a computer-based inhibitory control training programme enhanced by transcranial direct current stimulation (tDCS) and at a 6-week follow-up. At both assessments, an experimental VR paradigm was conducted and patients were characterized concerning eating disorder psychopathology, eating behaviour, general impulsivity and food craving. In the experimental task, one of two simultaneously presented objects (food vs. office tools) had to be collected. Food was recognized faster than office tools and subsequent approach behaviour was initiated faster, whereas thereafter, food was collected slower than office tools. Exploratory, we could not find a modulatory effect of applied tDCS on the interaction with food. No relationship between behavioural biases and sample characterizations could be detected. Two different stages in the manual interaction with food were found: a faster first stage that comprises recognition and movement initiation and a slower second stage that comprises controlled handling and may reflect aversive motivational processes. As the behavioural patterns do not change with an ameliorated BED-psychopathology at the second assessment, the task seems insensitive in detecting translational interconnections between behavioural biases and BED-characteristics.

Level of evidence: Level I, experimental study.

**Supplementary Information:**

The online version contains supplementary material available at 10.1007/s40519-023-01571-2.

## Introduction

Since 2013, Binge Eating Disorder (BED) is a distinct eating disorder according to the Fifth Edition of the Diagnostic and Statistical Manual of Mental Disorders (DSM-5). Diverse studies investigated underlying cognitive mechanisms, and their role in the development, maintenance as well as remission of BED [1, 2]. Furthermore, especially if disorder-relevant stimuli (e.g., food) are involved, cognitive functions such as inhibition, working memory, and memory are impaired on a behavioural and neurophysiological level, indicating an association between BED and disorder-specific difficulties in cognitive domains and highlighting the relevance of addressing impaired cognitive functions in appropriate interventions in BED [3]. However, data on aspects of embodiment as reflected, for instance, by the specific dynamics of physical interaction with food stimuli, is scarce.

A core characteristic of BED is the recurrence of binge eating episodes at least once a week on average within the last 3 months and loss of control without inappropriate compensatory behaviours (e.g., fasting, purging, excessive exercise). According to the DSM-5 three of the following symptoms have to occur during the above-mentioned core symptom: eating faster than usual; eating until feeling uncomfortably full; eating large amounts of food when not physically hungry; eating alone because of being embarrassed by how much one is eating; feeling disgusted with oneself, depressed or very guilty after overeating. Last, people should report distress regarding their binge eating behaviour. Two theoretical frameworks seem suitable to explain the core symptom of BED. First, a dual-process system consisting of an impulsive system and a reflective system [4, 5]. The impulsive system mainly evaluates environmental cues regarding emotional and motivational relevance, whereas the reflective system comprises mainly deliberate processes such as cognitive control which also considers long-term consequences. Especially, in BED, a stronger impulsive system and a weaker reflective system leads to increased impulsivity and poor cognitive control towards food stimuli [3, 6, 7]. Another framework highlights food-related impulsivity as a central factor in BED: increased sensitivity to rewarding stimuli, increased rash and spontaneous behaviour towards rewarding stimuli or slower disentanglement of those [8, 9]. In sum, attentional biases for food indicate faster processing of food in an early orientation reaction and slower disengagement from these stimuli in patients with BED, thus suggesting to interfere with response inhibition and goal-directed behaviour [10–15]. In this context, a systematic review emphasizes impulsivity as a very heterogenous concept with multiple facets consisting of temporal impulsivity, motor impulsivity, reflection impulsivity, attentional lapses and risk taking, which all might be modulated by emotional and physiological states and activate different neural circuits and brain regions differently [16].

Especially, if food stimuli are involved, research has shown that several cognitive domains in patients with BED are altered compared to healthy controls: decreased mental flexibility [17], impaired response inhibition [11, 18–20], reduced visual disengagement from food [14, 21], decreased cognitive control [22] and elevated rapid orientation towards food [13, 15]. These altered cognitions were shown to be associated with the severity of BED symptoms [12, 23], Body-Mass-Index (BMI) [24] or general impulsivity [25]. Furthermore, reduction of these attentional biases by application of a inhibitory control training or a psychotherapy intervention showed also a reduction in eating disorder symptoms and food craving, highlighting the strong interconnection between psychopathology and cognition [26–28]. Investigating cognitive processes seem promising in finding causal links between the psychopathology and symptoms that can be operationalized in experimental paradigms. Recent work has also shown that BED might not be only different compared healthy controls, but also compared to other eating disorders in terms of the upcoming term of food addiction [29]. In this meta-analysis it is argued that BED is an overlapping construct with the newly upcoming construct of food addiction, interpreting the overeating rather a pattern of compulsive behaviour and highlighting the behavioural role of food addiction in the BED. Therefore, it could make sense to investigate the tight interconnection between behaviour and underlying cognitive processes.

Whereas most experimental paradigms measure exclusively cognitive domains, virtual reality (VR) in combination with motion tracking allows to investigate more complex appetitive behaviour such as grasping movements and their hypothesized underlying cognitive processes. In this context, embodied cognitions play a central role. Within the concept of embodied cognitions it is assumed, that the agent’s physical body causally contributes to cognitive processes [30]. Therefore, a link between behavioural patterns and underlying cognitive processes can be made. Previously it could be shown, that in a non-clinical population food stimuli is more prominent in the manual interaction than the control stimuli and this effect is more prominent in the VR compared to a 2D-touchscreen application, supporting the hypothesis of embodied cognitions [31, 32]. In the study of Max et al. [29], the participants had to detect and collect the target out of two concurrently presented stimuli in a binary-choice–forced-choice paradigm. Food was detected faster than office tools but collected slower afterwards. A faster approach movement was initiated after the detection, highlighting the interconnection between behavioural patterns and underlying processes. Stronger behavioural control towards food was observed, and this was associated with an increased activity in the right dorsolateral prefrontal cortex (dlPFC). Furthermore, VR allows a trade-off between lifelike manual interaction with stimuli and the possibility of an experimental-controlled environment, where different variables can be manipulated to the exact same extent within the different experimental conditions.

To validate and explore the clinical implication of these findings, we set out to investigate the VR paradigm in a sample with BED. We again use high-calorie food to achieve clearer effects when comparing to the control objects as we did in Max et al. (2021), as it was shown that high-calorie food displays differences to control objects better than low-calorie food [33]. Patients underwent the binary-choice–forced-choice paradigm at two times: before the participation in a clinical randomised controlled trial (RCT) and 4 weeks after finishing the RCT. In this RCT, patients with diagnosed BED underwent six sessions of a inhibitory control training to increase inhibitory control towards food in an antisaccade paradigm which was combined either with sham or 2 Milliampere (mA) verum stimulation using transcranial direct current stimulation (tDCS) [34]. The clinical effects of tDCS combined with an inhibitory control training on BED symptoms and a critical reflection of the obtained results are presented in detail in Giel et al. [34]. In the current project, we explore the differential effects in the manual interaction with food in the VR in a repeated-measure design. Because of its salient and rewarding nature especially in patients with BED, we expect only faster movement initiation towards food than towards office tools at the first measurement [35–37]. Furthermore, as BED is characterized by reduced cognitive control and increased impulsive eating behaviour, we expect faster handling of food than of office tools at the first measurement. After the inhibitory control training and the associated expected improvement in eating disorder pathology, this behavioural pattern should change at the second measurement, resulting in slower, thus less impulsive interaction with food. Past research already showed that an inhibitory training ameliorated attentional biases [26–28]. If there is a close interconnection between behavioural patterns and underlying cognitive processes, the behavioural patterns should also change. Exploratory we investigate the effects of sham vs. verum stimulation in the handling of food compared to office tools. To explore the clinical implications of the task, we tested if the behavioural patterns in the interaction with food are linked with eating disorder psychopathology, general impulsivity, cognitive and behavioural domains of eating behaviour and food craving at the first measurement. If there is a causal connection between behavioural biases and the facets of psychopathology, impulsivity, eating behaviour and food craving, the behavioural biases should predict just those facets assessed either by self-reported questionnaires or semi-structured clinical interviews. Furthermore, if these behavioural patterns are contributing factors in the development and maintenance of BED, changed behavioural patterns at the second measurement in relation to the first measurement should predict the change of above-mentioned facets of psychopathology, impulsivity, eating behaviour and food craving at the second measurement in relation to the first measurement. Exploratory, we investigate the effects of tDCS in between the two assessments in the VR to account for possible confounding effects on behavioural markers in the manual interaction with food objects.

## Methods

### Participants

All patients had to fulfil the criteria for BED according to DSM-5 [38]. The first 32 patients who gave informed consent were recruited from a randomised controlled trial, where an inhibitory control training was combined with placebo-controlled 2 mA tDCS applied to the right dorsolateral prefrontal cortex [34]. This sample size was chosen in accordance with the pilot-study with healthy subjects [31]. From the first 32 patients, one patient was excluded as the patient dropped out during the intervention. Thus, 31 patients (24 women, M_age_ = 36.26, SD_age_ = 13.37, M_BMI_ = 35.02, SD_BMI_ = 9.72) completed both appointments of the study. Further detailed descriptive statistics can be looked up in Online Resource 4. 15 patients were randomly assigned to 2 mA verum tDCS, 16 patients received sham stimulation during the clinical intervention in between the two assessment points. No tDCS was applied during the assessment with the VR. Exclusion criteria were current or lifetime psychotic disorder, bipolar-I disorder, current substance dependence, current suicidality, previous bariatric surgery, severe somatic diseases which influence weight or eating behaviour and are not controlled by stable medication, severe neurologic disease and impaired vision, ametropia, eye diseases which prevent the execution of the task. For the completion of both study appointments the patients received 20€. The study was approved by the ethics committee of the Medical Faculty Tübingen (043/2020BO2), and all participants gave informed consent. The study was preregistered (https://doi.org/10.17605/OSF.IO/WN4U5).

### Apparatus

#### Virtual reality (VR)

The patients wore a head-mounted display (HMD) with continuous tracking of head rotation and interindividual adjusted inter-pupillary distance (Oculus Rift CV1; Oculus VR, Inc., Menlo Park, USA). Both screens of the HMD had a resolution of 1080 × 1200 pixels. The virtual environment was built and controlled by Unity 3D (5.6.2f1) with a bundled version of OVRPlugin. Trajectories of the patients’ hand were tracked by a near-infrared sensor (Leap Motion Inc., San Francisco, USA) and were streamed in real-time into the HMD. Thus, the patients could interact with the virtual stimuli by actual movements of their hand. The sensor was placed on a small table in front of the patients and covered a range of approximately 1600 cm^2^ of the patients’ dominant hand. The 3D-stimuli of a previous VR study by our work group were used [31]. The stimuli consisted of 48 different variations stemming from three categories (balls, food, office objects). For each category, there were four further sub-category (balls: handball, tennisball, beachball, baseball; food: cupcake, donut, pizza, burger; office objects: stapler, hole-puncher, folder, calculator). A full list of the different stimuli described can be found in Online Resource 5. Each stimulus was rated concerning valence, arousal, urge to grasp, aesthetics, subjective estimated size and comfort of grasping at the first and second experimental measure, see Online Resource 1 and Online Resource 2.

### Procedure

The study consisted of two measurements (*T0* and *T1*) with at least 6 weeks in between the appointments. In between the appointments the patients participated in a study examining the clinical effects of a tDCS-enhanced inhibitory control training on BED. The 2-week lasting inhibitory control training consisted of an antisaccade-task with food pictures, where the participants were instructed to look on the opposing direction, where the food picture was presented. Parallel to the training, tDCS was applied to the right dorsolateral prefrontal cortex. Further details can be looked up in the publication of Giel et al. [34]. T0 took place during the baseline-assessment of the tDCS-enhanced inhibitory control training, then the patients underwent the training within 2 weeks and 4 weeks after completion, *T1* took place. The procedure of both appointments was identical. Three hours prior to the experiment the patients were instructed to be fasted. At the experimental measure this was checked by self-report and each patient declared conformity with this instruction. Before the first appointment which mainly comprised the behavioural task in the VR, demographic data, weight and height measured on a scale, the diagnosis and severity of the BED examined by the Eating Disorder Examination (EDE; [39]), Three-Factor Eating Questionnaire (TFEQ; [40]), Barratt Impulsiveness Scale (BIS-15; [41]), UPPS Impulsiveness Scale (UPPS; [42]) were assessed at the baseline of the clinical trial which was conducted 1 to 14 days (*M*_days_ = 2.91; *SD*_days_ = 2.59) before the first appointment. At the second appointment, the above-mentioned data were assessed prior to the behavioural task in the VR. Food-Craving was measured by the Food-Craving Questionnaire-State [43] before and after the behavioural task.

**Fig. 1:**
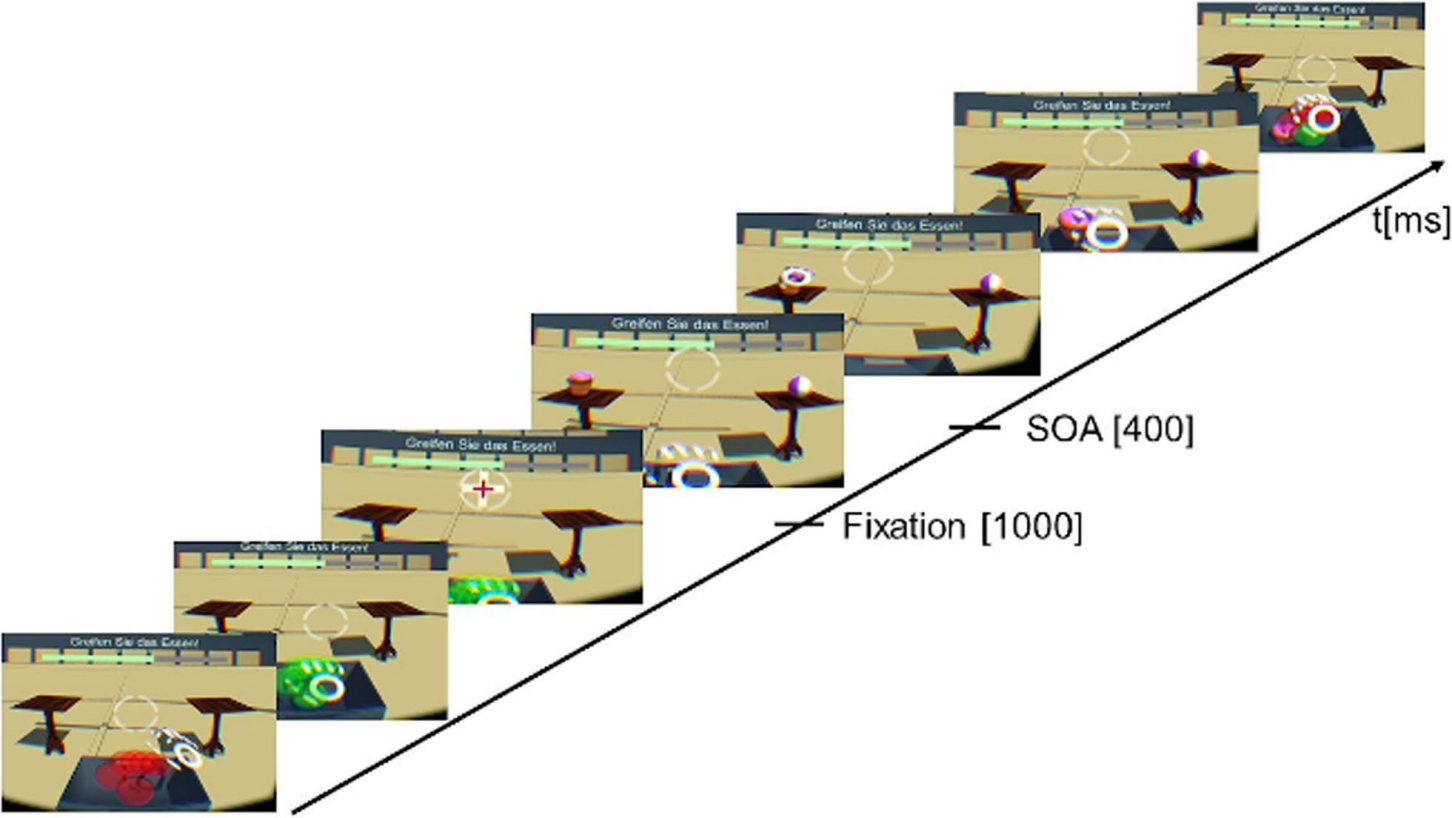
Visualization of a trial of the condition “food”. The target stimulus to be grasped was a chocolate cupcake with pink icing. The distractor stimulus was a beachball. After matching the initial hand pose with the standardized hand pose, the crosshair of the HMD had to be aligned with the fixation cross for 1 s. After 400 ms, target and distractor stimulus were presented on the left and right table. The image was previously used by Max SM, Schroeder PA, Blechert J, Giel KE, Ehlis A-C, Plewnia C (2021) Mind the food: behavioural characteristics and imaging signatures of the specific handling of food objects. Brain Structure and Function 226 (4):1169–1183

For the behavioural task in the VR, the patients were equipped with the HMD and completed practice trials to get familiar with operating the system. An exemplary trial is shown in Fig. 1. Patients had to put their dominant hand in a standardized start position to start a trial. The start position was marked by seven red coloured spheres which turned green if the hand was placed in the right position. Afterwards, the crosshair of the HMD had to be aligned with a fixation cross for at least 1 s. With a stimulus onset asynchrony of 400 ms, one target and one distractor stimulus were presented concurrently next to each other. If the patients moved their hand before the presentation of the stimuli, an error message was displayed. The positions of target and distractor stimulus were counterbalanced on the left and right table. A trial was finished when the target stimulus was grasped and placed inside the box. If the target stimulus was placed outside of the box, the wrong stimulus was grasped or the grasping took longer than 5 s, and error message was displayed and the trial was discarded. The spatial distance between start position and target stimulus was approximately 40 cm. The whole behavioural task consisted of six blocks with 32 trials each. In two blocks each a certain stimulus category had to be grasped according to the visually presented block instruction: food, balls or office tools. Each stimulus category was selected twice as a target and as a distractor and were paired with each other counterbalanced and within the stimulus category each variation of the stimulus category was presented randomized and balanced on the left and right table. Across patients block order was counterbalanced and patients were allocated randomized to a block order. In total, the task took about 15 min. After the behavioural task, each presented stimulus was rated. In total, each of the two appointments took about 60 min.

### Materials

#### Eating disorder examination (EDE)

The EDE is a structured clinical interview to examine various eating disorders (Binge-Eating Disorder, Bulimia Nervosa, Anorexia Nervosa) and is adapted to the criteria of the DSM-5 [39]. It allows the quantification of a specific eating disorder pathology. The EDE consists of four subscales (restraint, eating concern, weight concern, shape concern). Restraint comprises the attempt to restrict food intake and to diet. Eating concern is characterized by other abnormalities concerning eating-like intrusive thoughts concerning eating. Weight-related worries are depicted by the subscale weight concern, like the impact of weight on the self-worth. Shape concern is operationalized similar to weight concern. Furthermore, a total score indicates the severity of eating disorder pathology [39]. For each scale, a higher score indicates a higher markedness of the corresponding scale.

#### Food-craving questionnaire-state (FCQ-S)

The FCQ-S assesses the desire to eat a specific food as a state variable. 15 items subdivide into three subscales: intense desire to eat/loss of control, positive affect and hunger [43]. Furthermore, a total score operationalizes a current food craving. For each scale, a higher score indicates a higher degree of food craving.

#### Three factor eating questionnaire (TFEQ)

The TFEQ operationalizes eating behaviour on three scales: restraint, disinhibition and hunger. Restraint includes dieting and avoidance of high-calorie food. Disinhibition comprises habitual, emotional and situational susceptibility. Hunger captures internal and external processing of hunger cues [40]. A higher score indicates a higher markedness of the eating behaviour component.

#### Barratt impulsiveness scale (BIS-15)

The BIS-15 assesses impulsivity, the drive to act fast and thoughtlessly without taking negative consequences into account. The BIS-15 [41] comprises 15 items which subdivide into the subscale attentional impulsivity, motor impulsivity and non-planning impulsivity. For each scale, a higher score indicates a higher degree of impulsivity.

#### UPPS impulsiveness scale (UPPS)

The UPPS operationalizes impulsivity on four scales: urgency, lack of premeditation, lack of perseverance and sensation seeking. Urgency reflects the tendency to follow impulses evoked by negative affect. Premeditation captures the tendency to act deliberately and consciously. The ability to stay focussed on a challenging task is covered by perseverance. Sensation seeking refers to the tendency enjoying risky and exciting activities [42]. A higher score on each scale reflects a higher markedness of the different faces of impulsivity.

### Data analysis and statistics

#### Behavioural data analysis

For every statistical test, a significance level of 5% was used. To check for the efficacy of the training intervention, paired *t* tests were performed on the overall score of the EDE and frequency of binge eating episodes during the last 4 weeks, an indicator for the general eating disorder pathology. On a behavioural level, incorrect trials in the VR were excluded from all analyses (0.16%). Incorrect trials were defined as follows: for the movement onset, reaction times above 2000 ms and below 200 ms as well as reaction times above 4000 ms for the collection time were considered premature/incorrect as a result of visual inspection. Furthermore, values deviating more than 2.5 *SD* from individual cell mean were considered outlier responses (3.71%). We standardized the reaction times for the food- and office-objects in relation to the ball-objects to account for individual differences in motor grasping of grasp-affordant objects. For each trial the individual’s mean reaction time for ball objects was subtracted. Concerning the first hypothesis, we accounted for individual differences in grasping food stimuli using a linear mixed model approach. All linear mixed models were calculated by the *lme4-*package of R [44]. Log-likelihood-tests between a linear mixed model with the fixed effect and a linear mixed model without the fixed effect were conducted to determine significance. Three fixed effects were tested within the linear mixed model: time (*T0* vs. *T1*), the category of the target stimuli (*Food* vs. *Office tools*) and exploratory the stimulation condition (*sham* vs. *verum*). To test the contrasts within the levels of the fixed effects, the *lsmeans*-package of R was used, based on the method of the least-squares means and adjusted by Tukey method [45]. To estimate effect sizes for fixed effects f^2^ was used which can also be used in mixed linear models [46, 47].

For the second hypothesis, multivariate linear regression was conducted. An individual bias of the manual actions for each patient was calculated by the difference of the ball standardized reaction times of food objects and the ball standardized reaction times of office tools:

(RT_Office_ – RT_Ball_) – (RT_Food_ – RT_Ball_).

Those individual biases were used as a predictor variable for the different markers of eating disorder pathology (EDE), eating behaviour (TFEQ), food craving (FCQ-S) and impulsivity (BIS-15, UPPS) at *T0*.

For the third hypothesis, the approach of the second hypothesis was used. However, a difference score between *T0* and *T1* was calculated for the individual biases of the manual actions and the different markers of eating disorder pathology, eating behaviour, food craving and impulsivity. A negative difference score represents a decrease in the individual bias and the different markers.

## Results

### Change in eating disorder psychopathology

At *T1*, general eating disorder psychopathology (*M* = 2.04, *SD* = 0.85) was significantly lower than at *T0* (*M* = 2.61, *SD* = 0.89), *t*(30) = 5.41,* p* < 0.001, *d* = 0.65. The same applied to the frequency of binge eating episodes in the last 4 weeks. At *T1*, significantly fewer binge eating episodes (*M* = 6.26, *SD* = 6.66) were reported compared to *T0* (*M* = 17.35, *SD* = 10.74), *t*(30) = 5.53,* p* < 0.001, *d* = 1.24.

### Effects of food on different stages of manual action in VR

#### Movement onset

Including the category of target stimuli as a fixed effect in the mixed model leads to a significantly better model than the random intercept-only model, χ^2^(1) = 109.21, *p* < 0.001, R^2^ = 0.09. The movement onset for the food-objects as a target was significantly faster than for the office-tool-objects (*M* = 28.11 ms), *t* = 10.49, *p* < 0.001, *f*^*2*^ = 0.015.

**Fig. 2:**
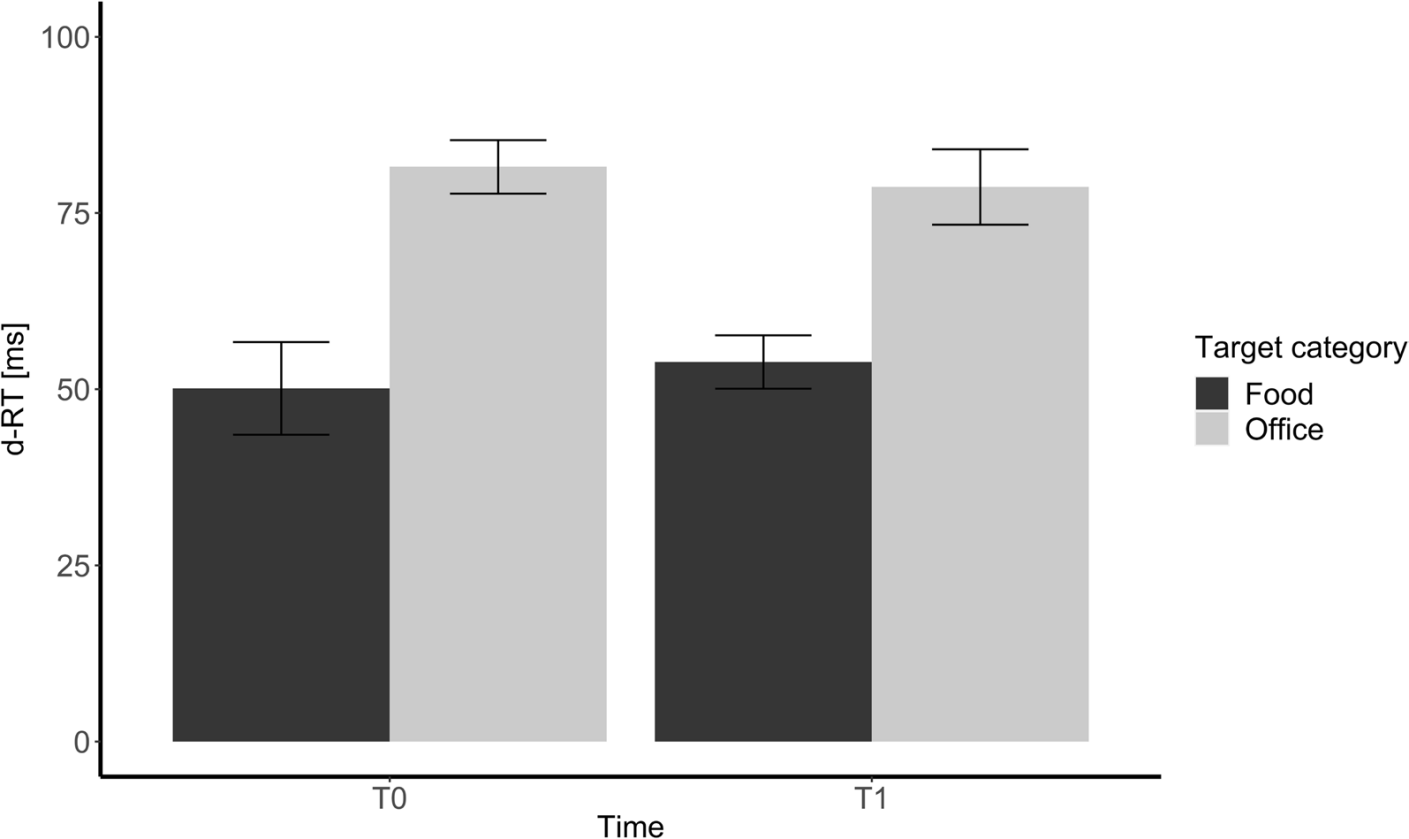
Movement onset in relation to the movement onset reaction times of the ball-objects. The bars represent the standard error. d-RT were calculated by subtracting the reaction time of the ball objects from the reaction time of the target objects

Including time as a fixed effects in the mixed model does not lead to a significantly better model than the random intercept-only model, χ^2^(1) = 0.02, *p* = 0.897, R^2^ = 0.08. In Fig. 2, the ball-object-corrected reaction times of the movement onset are depicted.

Exploratory, including stimulation as a fixed effect in the mixed model does not lead to a significantly better model than the random intercept-only model, χ^2^(1) = 0.78, *p* = 0.375, R^2^ = 0.08. For the raw reaction times of the movement onset, see Online Resource 3.

#### Collection time

**Fig. 3:**
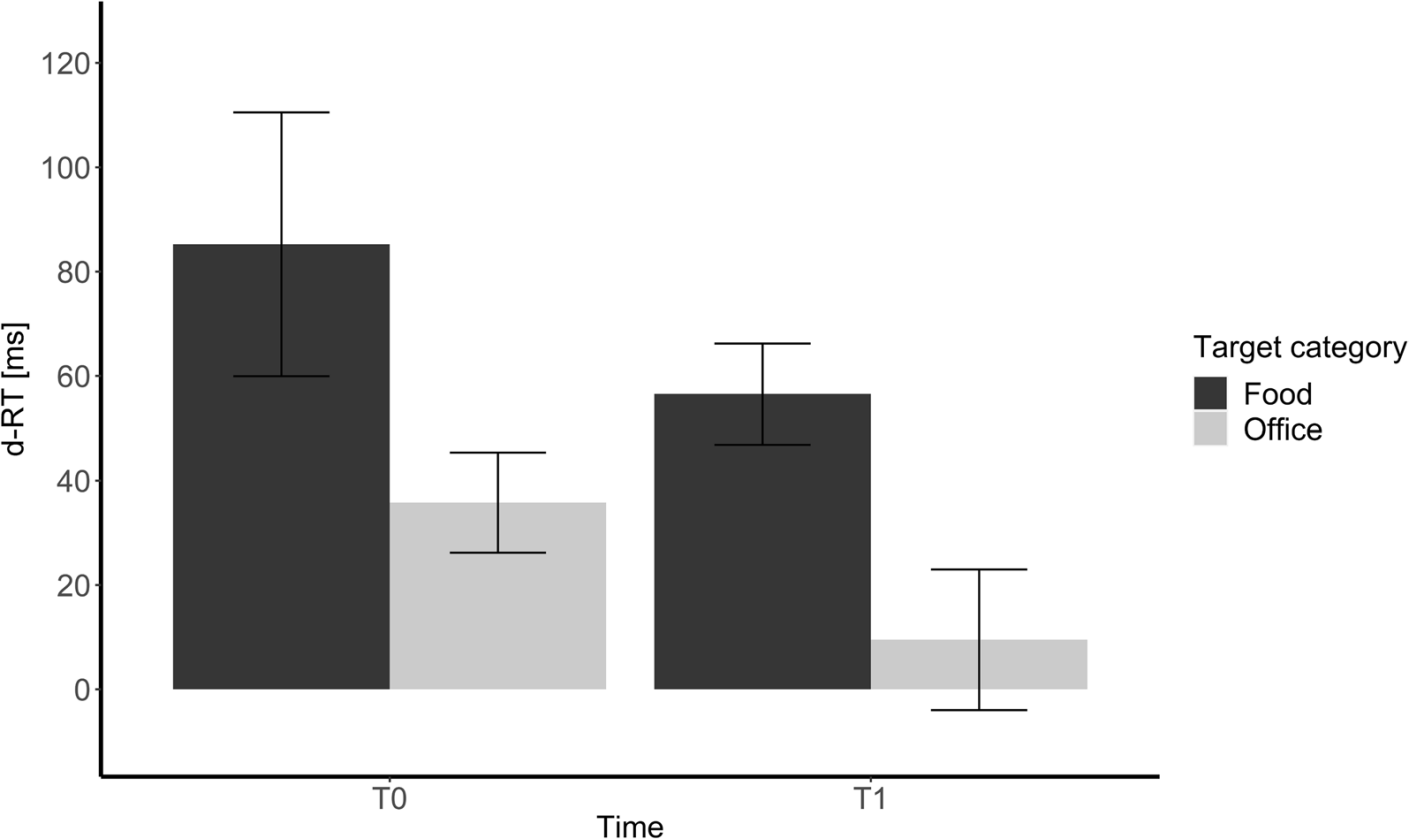
Collection time in relation to the movement onset reaction times of the ball-objects. The bars represent the standard error. d-RT were calculated by subtracting the reaction time of the ball objects from the reaction time of the target objects

Including the category of target stimuli as a fixed effect in the mixed model leads to a significantly better model than the random intercept-only model, χ^2^(1) = 50.48, *p* < 0.001, R^2^ = 0.19. Including the time as a fixed effect leads to a significantly better model than the random intercept-only model, χ^2^(1) = 49.27, *p* < 0.001, R^2^ = 0.11. Those two fixed effects do not interact with each other, as including two fixed effects as an interaction does not lead to a better model than two isolated fixed effects, χ^2^(1) = 0.03, *p* = 0.858, R^2^ = 0.19. Thus, food-objects were collected significantly slower than office-tool-objects (*M* = 48.10), *t* = 7.12, *p* < 0.001, *f*^*2*^ = 0.007. Furthermore, the collection time of the target stimuli was significantly faster at *T1* compared to T0 (*M* = 27.25), *t* = 4.02, *p* < 0.001, *f*^*2*^ = 0.003. As soon as the participants have left their initial hand position, they collected office-objects quicker than food-objects and an overall faster collection time from *T1* to *T0* irrespective of the target category could be observed. In Fig. 3, the ball-object-corrected reaction times of the collection time are depicted. Exploratory, including the stimulation as a fixed effect in the mixed model does not lead to a significantly better model than the random intercept-only model, χ^2^(1) = 0.03, *p* = 0.858, R^2^ = 0.18. For the raw reaction times of the collection time, see Online Resource 3.

### Individual bias of manual actions and markers of psychopathology, eating behaviour, food craving and impulsivity at *T*0

No markers concerning eating disorder psychopathology, eating behaviour or impulsivity at T0 were predicted by the behavioural patterns in the manual interaction with the food objects at T0. All estimates of the behavioural biases on the markers can be looked up in Table 1.

**Table 1:** Multiple linear regression of individual biases of manual actions on markers of psychopathology, eating behaviour, food craving and impulsivity at T0

	Movement onset bias				Collection time bias			
	*β*	*t*	*p*	*R*^2^	*β*	*t*	*p*	*R*^2^
FCQ total score	− 0.022	− 0.84	0.411	0.02	0.011	1.24	0.227	0.05
FCQ desire to eat/loss of control	− 0.010	− 0.88	0.384	0.03	0.006	1.38	0.178	0.06
FCQ reinforcement/positive affect	− 0.010	− 0.88	3.84	0.03	0.003	0.84	0.407	0.02
FCQ hunger	− 0.002	− 0.27	0.787	0.002	0.002	1.12	0.271	0.04
BIS total score	0.020	1.52	0.139	0.07	0.007	1.49	0.147	0.07
BIS non-planning Impulsivity	0.006	0.97	0.339	0.03	0.002	0.92	0.364	0.03
BIS motor impulsivity	0.007	1.30	0.203	0.06	0.003	1.71	0.099	0.09
BIS attentional impulsivity	0.007	1.18	0.248	0.05	0.002	0.8	0.424	0.02
EDE total score	− 0.002	− 0.93	0.363	0.03	0.001	1.08	0.287	0.04
EDE restraint	0.000	0.22	0.830	0.001	0.001	1.04	0.308	0.04
EDE eating concern	0.001	0.26	0.800	0.002	0.001	1.29	0.208	0.05
EDE weight concern	− 0.002	− 1.01	0.322	0.03	0.0002	0.33	0.746	0.004
EDE shape concern	− 0.005	− 1.96	0.059	0.12	0.001	0.63	0.533	0.01
EDE number of binge eating episodes during the last 4 weeks	− 0.039	− 2.00	0.055	0.12	− 0.005	− 0.74	0.467	0.02
TFEQ restraint	0.004	0.49	0.628	0.01	− 0.0003	− 0.10	0.918	0.0003
TFEQ disinhibition	0.002	0.52	0.609	0.01	0.003	1.74	0.096	0.10
TFEQ hunger	− 0.006	− 1.06	0.296	0.04	0.001	0.33	0.745	0.003
UPPS urgency	0.001	1.10	0.282	0.04	0.0002	0.55	0.586	0.01
UPPS lack of premeditation	0.002	1.87	0.072	0.11	0.0004	1.40	0.173	0.06
UPPS lack of perseverance	0.002	1.47	0.153	0.07	− 0.001	1.72	0.096	0.10
UPPS sensation seeking	− 0.002	− 1.35	0.186	0.06	− 0.001	− 1.92	0.065	0.11
BMI	− 0.017	− 0.93	0.361	0.03	− 0.002	− 0.33	0.744	0.004

*β* = Estimate of the marker on the behavioural bias; *t *values; *p* values; *R*^2^ = coefficient of determination. *FCQ* = Food-Craving-Questionnaire; *BIS* = Barratt-Impulsiveness Scale; *EDE* = Eating Disorder Examination; *TFEQ* = Three-Factor-Eating-Questionnaire; *UPPS* = UPPS impulsive behavior scale; *BMI* = Body-Mass-Index

### Changes of individual biases of manual actions and changes of markers of eating behaviour, food craving and impulsivity

No changes in the markers concerning eating disorder psychopathology, eating behaviour or impulsivity at *T1* in relation to *T0* were predicted by the changes of behavioural patterns in the manual interaction with the food objects at *T1* in relation to *T0*. All predictors of the multiple linear regression are listed in Table 2.

**Table 2:** Multiple linear regression of changes concerning individual biases of manual actions on changes in markers of psychopathology, eating behaviour, food craving and impulsivity

	ΔMovement onset bias				ΔCollection time bias			
	*β*	*t*	*p*	*R*^2^	*β*	*t*	*p*	*R*^2^
**Δ**FCQ total score	− 0.031	− 1.08	0.290	0.04	− 0.001	− 0.11	0.911	0.0004
**Δ**FCQ desire to eat/loss of control	− 0.015	− 1.06	0.296	0.04	− 0.002	− 0.047	0.639	0.008
**Δ**FCQ reinforcement/positive affect	− 0.018	− 0.156	0.130	0.08	− 0.002	− 0.47	0.645	0.01
**Δ**FCQ hunger	0.003	0.45	0.657	0.006	0.003	1.33	0.193	0.06
**Δ**BIS total score	− 0.006	− 0.40	0.695	0.01	0.004	0.95	0.350	0.03
**Δ**BIS non-planning impulsivity	− 0.005	− 0.65	0.523	0.01	0.004	1.66	0.109	0.08
**Δ**BIS motor impulsivity	− 0.003	− 0.43	0.672	0.01	− 0.004	− 1.75	0.092	0.10
**Δ**BIS attentional impulsivity	0.002	0.24	0.816	0.001	0.004	1.19	0.243	0.05
**Δ**EDE total score	− 0.0001	− 0.03	0.998	0.0001	0.0002	0.51	0.612	0.01
**Δ**EDE restraint	0.002	0.76	0.456	0.02	− 0.00003	− 0.04	0.971	0.00004
**Δ**EDE eating concern	0.0004	0.13	0.896	0.001	0.001	0.82	0.422	0.02
**Δ**EDE weight concern	− 0.001	− 0.36	0.719	0.004	− 0.0002	− 0.41	0.686	0.01
**Δ**EDE shape concern	− 0.002	− 0.84	0.407	0.02	0.0004	0.63	0.533	0.01
**Δ**EDE number of binge eating episodes during the last 4 weeks	− 0.041	− 1.59	0.122	0.08	0.001	0.24	0.812	0.002
**Δ**TFEQ restraint	− 0.009	− 0.86	0.396	0.02	− 0.004	− 1.16	0.255	0.04
**Δ**TFEQ disinhibition	0.007	1.00	0.325	0.03	0.0005	0.21	0.838	0.001
**Δ**TFEQ hunger	− 0.004	− 0.72	0.478	0.02	− 0.001	− 0.28	0.779	0.003
**Δ**UPPS urgency	− 0.001	− 1.25	0.220	0.05	− 0.10	− 1.81	0.081	0.10
**Δ**UPPS lack of premeditation	0.001	1.62	0.116	0.08	0.0004	0.00	1	< 0.0001
**Δ**UPPS lack of perseverance	0.0001	0.06	0.951	0.0001	0.0001	0.26	0.798	0.002
**Δ**UPPS sensation seeking	− 0.002	− 0.25	0.801	0.002	− 0.0001	− 0.44	0.660	0.007
**Δ**BMI	− 0.0001	− 0.06	0.955	0.0001	0.0003	0.47	0.643	0.008

*β* = Estimate of the marker on the behavioural bias; *t *values; *p* values; *R*^2^ = coefficient of determination. *FCQ* = Food-Craving-Questionnaire; *BIS* = Barratt-Impulsiveness Scale; *EDE* = Eating Disorder Examination; *TFEQ* = Three-Factor-Eating-Questionnaire; *UPPS* = UPPS impulsive behavior scale; *BMI* = Body-Mass-Index

## Discussion

The present study revealed differential effects in the manual interaction with food in VR in a clinical sample with BED. In line with our hypothesis, we found faster movement initiation towards food than towards office tools at the first measurement. In contradiction to our expectation, we did not observe faster handling of food than of office tools, as office tools were actually collected faster than food, consistent with the finding in healthy subjects [31]. Exploratory, we could not find a modulatory effect of applied tDCS in between the assessments in the VR on the manual interaction with food. Furthermore, even if the BED psychopathology of the patients significantly decreased from *T0* to *T1*, the behavioural patterns in the interaction with food compared to office tools did not change. At *T1*, still a significantly faster movement initiation towards food than towards office tools could be observed, whereas office tools were still collected faster than food. No link between markers of eating disorder pathology, impulsivity, eating behaviour, food craving and behavioural patterns in the interaction with food reached statistical significance.

A central finding is the faster movement initiation towards food than towards office tools. This first stage of manual interaction comprises a complex interplay between motoric and cognitive systems. On one hand, out of two concurrently objects the target has to be recognized and categorized, on the other hand a goal-directed behaviour towards the target has to be initiated. This is in line with the assumption, that especially in BED, strong impulsive responses towards food stimuli can be observed and those stimuli are processed faster than other stimuli [13, 15]. Thus, predisposed impulsive behaviour coupled with faster processing of food stimuli could be the factor, that leads to faster movement initiation towards food also in this new paradigm in the VR. Although it has to be interpreted with caution as higher levels of impulsivity within the sample did not lead to faster movement initiation towards food. Surprisingly, in contradiction of the assumption of generally elevated food-related impulsivity in patients with BED, food was collected slower than office tools after an approach movement was initiated [8, 9]. Even though this finding is unexpected, it coincides with the finding of the study of Max et al. with healthy participants. In this study, a slower collection time of food compared to office tools could be observed. It was hypothesized, that the slower collection time may reflect a more cautious handling due to increased personal relevance. The slower collection time could also mirror a certain ambivalence towards food experienced by patients with BED as it has been previously shown in terms of an approach–avoidance–conflict using psychophysiological measures [48, 49]. Alternatively, it may be the case, the patients in this study also wanted to make sure, that the highly hedonic stimuli are collected cautiously without getting lost. As the target had to be identified explicitly and may be categorized as a problematic object, an aversive motivational process may impede a fast collection.

Concerning behavioural patterns in the manual interaction with food, we tried to identify differential effects in the manual interaction due to generally changed psychopathology of the BED due to the participation in a treatment trial. In fact, the general eating disorder pathology as well as the frequency of binge eating episodes significantly decreased from *T0* to *T1* after the inhibitory control training. Even if the psychopathology changed, no changes in the behavioural pattern in the manual interaction with food could be observed, as no interaction effect became evident. The behavioural patterns at *T1* were comparable to the behavioural patterns at *T0*: in the first stage of manual interaction, compared to office tools, the categorization of food and movement initiation towards it was faster, whereas in the second stage, food was collected slower than office tools. Alternatively, it could be hypothesized that behavioural patterns are not changed due to rather short-term changes in psychopathology and that the underlying food- and eating-related cognitive processes remain rather unmodified. Thus, maybe behavioural patterns can hint at possible risk factors for long-term maintenance of remission in the BED.

To have a closer look into the interconnection between psychopathology of the BED and behavioural patterns in the manual interaction with food, we ran a multiple regression with markers of psychopathology, eating behaviour, food craving and impulsivity. The interconnection between the psychopathology and behavioural pattern in the experimental VR task seems to be rather scarce as we did not find any statistical significant prediction of the markers of psychopathology, eating behaviour, food craving and impulsivity by behavioural biases in the interaction with food. Using a repeated-measure within-subject design we were able to have an insight into the impact of changed behavioural markers in the interaction with food on altered markers of eating disorder psychopathology, impulsivity, food craving and eating behaviour on an individual level. Still, we were not able to find an association between changes on a behavioural level and changes on an eating disorder pathology level. This contradicts the assumption, that there is a strong interconnection between markers of psychopathology and task performance in a laboratory setting [26, 27]. As there is a great variety of experimental tasks used and the tasks greatly differ from each other, the task used in the present study seems to be inappropriate to uncover this interconnection. Especially in the domain of impulsivity the lacking connection to the behavioural patterns could be explained by the great heterogeneity of impulsivity itself. A mismatch of operationalized impulsivity on a behavioural level with the operationalized impulsivity on the level of the self-reported questionnaires might be the cause for that. Taken together, the findings concerning the clinical implications of the task used in the current study may be supported by studies, that emphasize the missing validity between laboratory tasks and self-report instruments [50]. Already in well-established experimental paradigms, a missing interconnection between laboratory task performance and general eating disorder pathology or trait impulsivity could be observed [51].

### Strengths and limits

One strength of this pilot study is the repeated-measure within-subject design, assessing the subjects before and after a clinical intervention, which ameliorated eating disorder psychopathology of BED. Thus, we could have drawn precise conclusions concerning contributing factors in the development, maintenance or remission of BED and behavioural patterns in a laboratory task in the VR. Findings of a repeated measure within a person in a state of clinical relevant BED compared to ameliorated psychopathology give a deeper insight into possible underlying mechanisms than comparing it to a matched control group. Another strength of the study is the usage of VR, which allows to investigate manual interaction with food in an experimentally controlled environment. Thus, a more ecologically valid situation could be created, where patients could interact with food using their actual hands instead of the commonly used button presses on keyboards. To make effects of VR even more present and the user experience even more immersive, it might be promising to include more modalities that occur within the interaction of food, like tactile feedback and smell. This could be achieved by standardized food prepared in a teaching kitchen and different objects placed at the position of the target objects to give tactile feedback. Last, the findings of the pilot-study concerning the behavioural patterns in the manual interaction with food could be replicated, emphasizing that this newly paradigm captures two different stages. A fast, impulsive first stage, where food is recognized faster and consequently movement towards food is initiated faster, and a slower, more controlled second stage, where food is collected slower and more cautiously. In this connection, the new paradigm does not seem to be sensitive to specific symptoms of BED, as the several assessed markers were not predicted by the behavioural biases in the interaction with food. Furthermore, healthy participants showed a similar behavioural pattern in the interaction with food as the patients with BED, even if a statistical comparison between the two groups would not be reasonable due to differences in the design, programming of the task and missing demographic matching between the two groups [31]. Nonetheless, even if there is an absence of significant effects between the clinical markers of BED and the behavioural patterns in the VR, discrete or more complex interconnections cannot be excluded as the sample size was rather small and many associations reached trending statistical significance. Especially concerning the construct of impulsivity, the used experimental paradigm mismatched with the impulsivity-related self-reported questionnaires, highlighting a more cautious selection of different approaches in operationalizing impulsivity in future studies. Future research using VR to explore the manual interaction with food and its clinical implications may profit from focusing on underlying neurophysiological processes to have a multimethod approach on assessing psychopathology-related biases using neuroimaging and neuromodulatory techniques as it may seem promising to find connections between clinical markers of BED-like impulsivity and responses of the prefrontal cortex [52].

## What is already known on this subject?

Several frameworks propose the existence of a dual-process system consisting of an impulsive and a reflective system. Especially in the field of BED cognitive mechanisms seem altered when food stimuli are involved, such as decreased mental flexibility [17], impaired response inhibition [11, 18–20], reduced visual disengagement from food [14, 21], decreased cognitive control [22] and elevated rapid orientation towards food [13, 15]. These altered cognitions were shown to be associated with the severity of BED symptoms [12, 23], Body-Mass-Index (BMI) [24] or general impulsivity [25] thus allowing making a link between eating-disorder pathology and the underlying eating-disorder-specific cognitions.

### What this study adds?

In sum, this study could demonstrate two different stages in the manual interaction with food in patients with BED and replicate a behavioural pattern that has been found in a previous study with healthy participants: a first stage, where food as a target is recognized faster and movement towards food is initiated faster than for office tools and a second stage, where food is collected slower than office tools, thus reflecting more cautious handling which might be explained by ambivalence or aversive motivational processes like avoidance. However, this behavioural pattern does not change with an ameliorated psychopathology of BED. Therefore, this paradigm does not seem sensitive enough to detect interconnections between eating disorder pathology, impulsivity, eating behaviour, food craving and the behavioural patterns in the VR. More insight into underlying motivational processes in the manual interaction with food and its underlying neurophysiological processes is needed to draw a more overall picture on the associations between neurophysiological, clinical and behavioural markers in the BED.

## Supplementary Information

Below is the link to the electronic supplementary material.

Supplementary file1 (PDF 24 KB)

Supplementary file2 (PDF 15 KB)

Supplementary file3 (PDF 15 KB)

Supplementary file4 (PDF 101 KB)

Supplementary file5 (PDF 102 KB)

## Data Availability

The data sets generated during and/or analysed during the current study are available from the corresponding author on reasonable request.
