# Supplementary material for: Behavioural biases in the interaction with food objects in virtual reality and its clinical implication for binge eating disorder
Source: Eat Weight Disord. 2023 May 24;28(1):46. doi: 10.1007/s40519-023-01571-2 (PMC10209312; doi:10.1007/s40519-023-01571-2)
Supplement: Supplementary file 1 — Supplementary file1 (PDF 24 KB) [file 40519_2023_1571_MOESM1_ESM.pdf]

Online Resource 1

(Behavioural biases in the interaction with food objects in virtual reality and its clinical implication for binge eating disorder; Eating and Weight Disorders – Studies on Anorexia, Bulimia and Obesity; Max, Schag, Giel, Plewnia; University Hospital Tübingen, Tübingen Center for Mental Health, Department of Psychiatry and Psychotherapy, Neurophysiology & Interventional Neuropsychiatry, Calwerstraße 14, 72076 Tübingen – Germany, christian.plewnia@med.uni.tuebingen.de)

Ratings of VR stimuli at T0

Stimulus	Mean Valence (SD)	Mean Arousal (SD)	Mean Urge to grasp (SD)	Mean Aesthetics (SD)	Mean subjective Size (SD)	Mean Grasp Comfort (SD)
**Baseball**	**57.67 (16.50)**	**47.89 (18.52)**	**54.6 (18.20)**	**57.67 (16.50)**	**48.51 (18.01)**	**65.14 (16.84)**
Baseball_1	61.58 (16.93)	47.19 (19.20)	54.87 (18.37)	61.58 (16.93)	51.16 (20.71)	63.58 (18.23)
Baseball_2	55.77 (22.72)	48.23 (25.21)	55.26 (23.11)	55.77 (22.72)	47.06 (21.14)	65 (21.78)
Baseball_3	57.1 (20.48)	46.74 (21.47)	54.35 (20.53)	57.1 (20.48)	50.68 (22.14)	66.26 (18.01)
Baseball_4	56.23 (17.12)	49.39 (21.74)	53.9 (20.37)	56.23 (17.12)	45.13 (17.56)	65.71 (18.57)
**Beachball**	**52.94 (15.35)**	**46.52 (16.99)**	**54.73 (20.59)**	**52.94 (15.35)**	**57 (18.89)**	**63.57 (20.03)**
Beachball_1	56.48 (18.48)	47.74 (20.15)	60.26 (24.51)	56.48 (18.48)	59.55 (21.66)	63.68 (21.73)
Beachball_2	51.55 (19.85)	47.52 (22.41)	53.84 (24.90)	51.55 (19.85)	57.1 (23.31)	66.81 (24.45)
Beachball_3	55.32 (23.62)	46.71 (21.94)	53.87 (21.78)	55.32 (23.62)	54.87 (20.92)	64 (22.89)
Beachball_4	48.42 (18.26)	44.1 (20.05)	50.94 (24.90)	48.42 (18.26)	56.48 (20.64)	59.81 (22.91)
**Handball**	**53.92 (15.02)**	**49.74 (20.09)**	**54.84 (19.03)**	**53.92 (15.02)**	**59.46 (16.76)**	**65.23 (19.19)**
Handball_1	57.81 (20.89)	52.03 (23.08)	56.42 (19.95)	57.81 (20.89)	61.03 (16.25)	66.42 (17.40)
Handball_2	43.42 (24.57)	46.74 (25.16)	50.9 (21.74)	43.42 (24.57)	60.48 (18.86)	62.61 (22.39)
Handball_3	57.19 (22.22)	53.03 (22.20)	55.13 (23.01)	57.19 (22.22)	59.55 (20.19)	63.97 (23.49)
Handball_4	57.26 (19.88)	47.16 (22.82)	56.9 (23.20)	57.26 (19.88)	56.77 (20.80)	67.94 (21.71)
**Tennisball**	**57.79 (13.28)**	**47.53 (20.84)**	**57.23 (21.46)**	**57.79 (13.28)**	**48.23 (20.14)**	**65.45 (18.83)**
Tennisball_1	55.81 (22.07)	48.71 (22.31)	63.26 (23.08)	55.81 (22.07)	49.32 (22.37)	67.32 (19.68)
Tennisball_2	61.42 (19.15)	48.45 (26.75)	56.81 (25.25)	61.42 (19.15)	46.32 (21.48)	65.06 (21.12)
Tennisball_3	57.35 (18.90)	42.97 (23.23)	55.48 (23.56)	57.35 (18.90)	48.32 (23.74)	67.03 (21.85)
Tennisball_4	56.58 (20.50)	50 (25.42)	53.35 (27.05)	56.58 (20.50)	48.97 (21.24)	62.39 (21.84)
**Burger**	**53.87 (19.49)**	**53.68 (15.22)**	**58.48 (19.92)**	**53.87 (19.49)**	**58.33 (15.29)**	**60.27 (18.28)**
Burger_1	47.84 (26.41)	52.35 (24.87)	58.19 (23.14)	47.84 (26.41)	58.45 (21.64)	62.19 (22.59)
Burger_2	55.16 (23.02)	52.03 (19.42)	58.48 (20.82)	55.16 (23.02)	57.32 (19.07)	58.74 (21.67)
Burger_3	63.26 (18.31)	60.23 (16.91)	64.58 (22.59)	63.26 (18.31)	62.52 (14.02)	65.52 (18.31)
Burger_4	49.23 (24.94)	50.1 (25.73)	52.68 (27.62)	49.23 (24.94)	55.03 (22.49)	54.61 (24.27)
**Cupcake**	**63.51 (15.91)**	**59.08 (17.18)**	**59.26 (21.08)**	**63.51 (15.91)**	**52.31 (17.03)**	**63.52 (17.54)**
Cupcake_1	66.35 (16.51)	60.26 (20.88)	62.29 (24.58)	66.35 (16.51)	53.29 (18.85)	61.65 (20.56)
Cupcake_2	64.74 (18.63)	61.32 (19.62)	58.03 (24.33)	64.74 (18.63)	53.87 (20.29)	66.06 (20.49)
Cupcake_3	59 (23.25)	55.71 (23.03)	57 (23.20)	59 (23.25)	53.52 (17.58)	63.9 (19.93)

Cupcake_4	63.94 (19.07)	59.03 (18.21)	59.71 (22.73)	63.94 (19.07)	48.55 (21.65)	62.48 (18.92)
**Donut**	**64.43 (16.36)**	**58.3 (16.86)**	**58.81 (21.78)**	**64.43 (16.36)**	**53.1 (17.61)**	**64.56 (19.17)**
Donut_1	61.77 (19.87)	54.03 (21.78)	54.84 (26.53)	61.77 (19.87)	50.74 (19.34)	65.1 (21.61)
Donut_2	62.35 (24.51)	56.03 (22.39)	57.81 (25.78)	62.35 (24.51)	52.61 (21.56)	63.58 (22.96)
Donut_3	65.55 (17.11)	62.06 (18.70)	62.48 (22.13)	65.55 (17.11)	53.65 (20.76)	62.03 (23.28)
Donut_4	68.03 (17.56)	61.06 (19.32)	60.13 (24.83)	68.03 (17.56)	55.42 (21.52)	67.52 (16.78)
**Pizza**	**59.14 (18.26)**	**58.31 (13.13)**	**62.35 (16.64)**	**59.14 (18.26)**	**65.94 (14.30)**	**62.56 (18.32)**
Pizza_1	61.19 (18.28)	59.26 (17.05)	62.58 (18.61)	61.19 (18.28)	66.39 (15.28)	63.06 (20.02)
Pizza_2	60.45 (22.18)	58.48 (15.03)	64.42 (19.72)	60.45 (22.18)	68.39 (13.93)	60.87 (20.53)
Pizza_3	54.32 (25.05)	55.94 (21.05)	61.1 (22.65)	54.32 (25.05)	63.16 (18.07)	61.81 (22.77)
Pizza_4	60.58 (19.51)	59.55 (14.81)	61.32 (16.73)	60.58 (19.51)	65.84 (17.57)	64.52 (20.13)
**Calculator**	**50 (16.43)**	**40.55 (19.68)**	**48.98 (20.01)**	**50 (16.43)**	**49.65 (15.95)**	**57.91 (18.31)**
Calculator_1	43.84 (23.96)	34.94 (23.19)	46.68 (24.98)	43.84 (23.96)	49.19 (18.09)	55.97 (20.18)
Calculator_2	58.9 (24.44)	49.61 (24.40)	51.16 (21.77)	58.9 (24.44)	52.77 (17.69)	58.61 (23.64)
Calculator_3	51.65 (22.87)	42.1 (21.68)	52.65 (25.39)	51.65 (22.87)	50.23 (20.76)	55.84 (23.41)
Calculator _4	45.61 (24.16)	35.55 (23.18)	45.42 (24.56)	45.61 (24.16)	46.39 (18.15)	61.23 (20.71)
**Folder**	**53.68 (15.49)**	**41.48 (18.16)**	**52.89 (21.19)**	**53.68 (15.49)**	**66.5 (15.26)**	**58.25 (20.82)**
Folder_1	46.32 (19.81)	35.13 (20.67)	48.68 (21.38)	46.32 (19.81)	65.35 (17.46)	56.19 (21.86)
Folder_2	60.32 (26.23)	46.06 (23.04)	55.9 (25.12)	60.32 (26.23)	67.06 (20.45)	56.35 (25.83)
Folder_3	56.9 (19.06)	44.32 (24.49)	54.45 (26.22)	56.9 (19.06)	66.1 (16.55)	62.42 (22.23)
Folder_4	51.16 (18.67)	40.42 (20.23)	52.52 (24.72)	51.16 (18.67)	67.48 (14.82)	58.03 (22.77)
**Hole-puncher**	**39.54 (15.35)**	**34.43 (16.71)**	**44.34 (19.54)**	**39.54 (15.35)**	**59.56 (12.52)**	**54.55 (17.40)**
Hole-puncher_1	41.1 (16.74)	35.42 (19.63)	45.55 (22.27)	41.1 (16.74)	57.61 (15.91)	52.29 (18.90)
Hole-puncher_2	41.68 (22.67)	36.97 (19.31)	43.52 (22.85)	41.68 (22.67)	62.39 (18.94)	56.48 (19.22)
Hole_puncher_3	40.71 (19.95)	33.48 (23.03)	43.81 (24.34)	40.71 (19.95)	58.19 (14.72)	55 (19.13)
Hole_puncher_4	34.68 (21.90)	31.84 (20.70)	44.48 (26.78)	34.68 (21.90)	60.06 (17.95)	54.42 (20.79)
**Stapler**	**45.85 (16.55)**	**40.72 (18.91)**	**47.15 (20.90)**	**45.85 (16.55)**	**48.09 (13.62)**	**54.46 (19.97)**
Stapler_1	44.13 (21.31)	39.45 (23.08)	48.26 (22.54)	44.13 (21.31)	46.65 (16.49)	54.55 (22.06)
Stapler_2	46.71 (20.43)	40 (21.93)	49.26 (24.16)	46.71 (20.43)	48.1 (17.34)	53.74 (22.00)
Stapler_3	46.97 (18.60)	43.06 (20.13)	44.32 (23.62)	46.97 (18.60)	48.58 (15.47)	55.35 (20.72)
Stapler_4	45.58 (21.09)	40.35 (20.45)	46.74 (24.84)	45.58 (21.09)	49.03 (19.53)	54.19 (22.98)

Note. Ratings on VR stimuli were reported on a visual analogue scale ranging from 0 to 100. 100 is reflecting a high score on the corresponding scale, whereas 0 is reflecting a low score. Mean ratings and standard deviations per category and per item are reported.
