# Supplementary material for: Behavioural biases in the interaction with food objects in virtual reality and its clinical implication for binge eating disorder
Source: Eat Weight Disord. 2023 May 24;28(1):46. doi: 10.1007/s40519-023-01571-2 (PMC10209312; doi:10.1007/s40519-023-01571-2)
Supplement: Supplementary file 2 — Supplementary file2 (PDF 15 KB) [file 40519_2023_1571_MOESM2_ESM.pdf]

Online Resource 2

(Behavioural biases in the interaction with food objects in virtual reality and its clinical implication for binge eating disorder; Eating and Weight Disorders – Studies on Anorexia, Bulimia and Obesity; Max, Schag, Giel, Plewnia; University Hospital Tübingen, Tübingen Center for Mental Health, Department of Psychiatry and Psychotherapy, Neurophysiology & Interventional Neuropsychiatry, Calwerstraße 14, 72076 Tübingen – Germany, christian.plewnia@med.uni.tuebingen.de)

Ratings of VR stimuli at T1

Stimulus	Mean Valence (SD)	Mean Arousal (SD)	Mean Urge to grasp (SD)	Mean Aesthetics (SD)	Mean subjective Size (SD)	Mean Grasp Comfort (SD)
**Baseball**	**53.46 (15.52)**	**46.26 (17.78)**	**50.7 (22.80)**	**53.46 (15.52)**	**48.33 (15.93)**	**62.04 (20.75)**
Baseball_1	58.03 (14.99)	47.81 (21.29)	49.58 (24.60)	58.03 (14.99)	50.29 (18.17)	64.71 (22.91)
Baseball_2	50.84 (22.49)	46.68 (22.74)	51.03 (24.74)	50.84 (22.49)	46.74 (18.84)	58.81 (22.45)
Baseball_3	49.45 (20.68)	43.03 (19.61)	52.48 (24.52)	49.45 (20.68)	47.87 (19.31)	61.74 (24.12)
Baseball_4	55.52 (17.49)	47.52 (20.07)	49.71 (24.44)	55.52 (17.49)	48.42 (18.44)	62.9 (21.88)
**Beachball**	**55.6 (16.91)**	**46.01 (18.20)**	**52.73 (23.26)**	**55.6 (16.91)**	**59.44 (14.92)**	**62.6 (22.84)**
Beachball_1	60.94 (20.44)	46.65 (21.96)	54.32 (25.27)	60.94 (20.44)	59.52 (17.31)	60.77 (24.05)
Beachball_2	54.94 (18.11)	44.45 (21.57)	50.42 (24.06)	54.94 (18.11)	60.55 (19.94)	63.87 (24.34)
Beachball_3	53.45 (20.23)	48.77 (18.76)	52.65 (22.75)	53.45 (20.23)	56.61 (18.59)	62.9 (23.80)
Beachball_4	53.06 (23.10)	44.16 (23.12)	53.55 (25.58)	53.06 (23.10)	61.06 (15.67)	62.87 (25.18)
**Handball**	**52.49 (17.64)**	**45.21 (19.42)**	**51.41 (21.95)**	**52.49 (17.64)**	**58.93 (12.13)**	**63.89 (21.16)**
Handball_1	50.97 (22.58)	45.74 (24.13)	54.39 (24.54)	50.97 (22.58)	58.74 (17.18)	64.55 (23.24)
Handball_2	48.94 (23.49)	40.03 (22.01)	50.13 (26.10)	48.94 (23.49)	59.06 (16.10)	62.61 (22.94)
Handball_3	56.1 (24.53)	49.03 (22.42)	49.48 (24.25)	56.1 (24.53)	57.87 (18.79)	65.58 (23.05)
Handball_4	53.97 (20.69)	46.03 (22.01)	51.65 (22.62)	53.97 (20.69)	60.03 (15.57)	62.81 (22.91)
**Tennisball**	**52.6 (13.79)**	**45.3 (19.06)**	**48.56 (22.36)**	**52.6 (13.79)**	**48.95 (16.13)**	**62.6 (19.13)**
Tennisball_1	49.13 (22.47)	41.77 (22.92)	47.39 (25.20)	49.13 (22.47)	43.03 (17.67)	59.32 (21.59)
Tennisball_2	54.77 (21.31)	47.29 (20.88)	47.68 (25.37)	54.77 (21.31)	47.65 (18.70)	60.65 (23.90)
Tennisball_3	57.84 (17.83)	47.42 (22.85)	50.97 (24.04)	57.84 (17.83)	54.68 (20.60)	68.29 (20.84)
Tennisball_4	48.65 (20.16)	44.71 (21.96)	48.23 (24.26)	48.65 (20.16)	50.45 (21.48)	62.13 (23.49)
**Burger**	**51.73 (18.32)**	**52.73 (18.73)**	**54.38 (22.39)**	**51.73 (18.32)**	**62.25 (14.08)**	**59.72 (20.09)**
Burger_1	51.97 (23.53)	53.23 (21.01)	52.42 (22.72)	51.97 (23.53)	60.84 (17.17)	60.42 (23.03)
Burger_2	53.16 (24.02)	51.16 (21.12)	54.65 (25.89)	53.16 (24.02)	61.77 (17.50)	59.61 (23.21)
Burger_3	52 (20.26)	52.35 (22.43)	56.71 (23.41)	52 (20.26)	62 (16.03)	62.16 (25.93)
Burger_4	49.77 (19.77)	54.16 (21.56)	53.74 (22.85)	49.77 (19.77)	64.39 (14.17)	56.68 (20.83)
**Cupcake**	**54.02 (19.43)**	**52.64 (18.71)**	**48.61 (22.63)**	**54.02 (19.43)**	**48.67 (14.21)**	**57.98 (21.36)**
Cupcake_1	56.65 (21.78)	53.48 (21.09)	49.29 (25.06)	56.65 (21.78)	48.52 (17.82)	59.26 (21.92)
Cupcake_2	54.19 (19.75)	50.68 (22.37)	46.19 (24.43)	54.19 (19.75)	49.45 (15.24)	58.32 (23.22)
Cupcake_3	49.45 (25.06)	48.45 (21.58)	47.61 (24.76)	49.45 (25.06)	47.97 (15.40)	56.48 (23.96)

Cupcake_4	55.77 (20.59)	57.94 (20.26)	51.35 (24.45)	55.77 (20.59)	48.74 (17.06)	57.87 (23.60)
**Donut**	**57.82 (19.92)**	**53.87 (20.06)**	**52.64 (23.69)**	**57.82 (19.92)**	**51.69 (12.87)**	**59.75 (20.70)**
Donut_1	54.61 (23.88)	52.74 (21.65)	51 (25.07)	54.61 (23.88)	51.1 (17.11)	58.81 (22.42)
Donut_2	55.29 (20.51)	52.71 (21.15)	50.23 (24.03)	55.29 (20.51)	50.84 (16.44)	57.26 (21.12)
Donut_3	63.16 (21.95)	56.29 (24.68)	56.26 (25.58)	63.16 (21.95)	50.77 (20.48)	61.06 (21.35)
Donut_4	58.23 (25.23)	53.74 (22.69)	53.06 (26.73)	58.23 (25.23)	54.06 (14.50)	61.87 (24.97)
**Pizza**	**51.6 (18.99)**	**51.44 (19.63)**	**53.21 (23.58)**	**51.6 (18.99)**	**62.35 (16.00)**	**57.3 (21.81)**
Pizza_1	53.48 (22.53)	51 (22.28)	56.35 (24.26)	53.48 (22.53)	64.55 (17.59)	57 (23.50)
Pizza_2	49.71 (24.30)	50.13 (22.90)	51.97 (26.33)	49.71 (24.30)	65.55 (19.91)	59.29 (26.27)
Pizza_3	49.48 (22.77)	50.16 (19.68)	50.55 (24.72)	49.48 (22.77)	59.97 (19.59)	53.61 (23.34)
Pizza_4	53.71 (19.28)	54.48 (21.40)	53.97 (26.36)	53.71 (19.28)	59.35 (15.60)	59.29 (21.21)
**Calculator**	**43.69 (16.17)**	**37 (16.18)**	**44.39 (19.58)**	**43.69 (16.17)**	**49.73 (13.58)**	**55.73 (20.35)**
Calculator_1	42.03 (20.82)	31.97 (17.55)	44 (23.49)	42.03 (20.82)	48.71 (14.18)	54.52 (22.16)
Calculator_2	45.39 (23.03)	39.77 (22.06)	46.35 (23.95)	45.39 (23.03)	50.94 (20.99)	58.58 (23.90)
Calculator_3	47 (18.51)	37.84 (20.82)	46.32 (21.75)	47 (18.51)	53.77 (16.64)	56.42 (26.13)
Calculator _4	40.35 (18.65)	38.42 (18.05)	40.87 (22.49)	40.35 (18.65)	45.52 (15.64)	53.42 (22.19)
**Folder**	**47.64 (14.77)**	**39.1 (18.50)**	**47.7 (22.47)**	**47.64 (14.77)**	**66.45 (15.00)**	**56.58 (23.11)**
Folder_1	45.68 (17.13)	35.87 (19.03)	45.84 (25.85)	45.68 (17.13)	64.71 (19.20)	56.55 (24.34)
Folder_2	50.48 (19.78)	42.06 (25.10)	47.58 (24.67)	50.48 (19.78)	69.61 (13.28)	56.06 (27.48)
Folder_3	47.03 (19.59)	39.1 (20.56)	46.77 (25.81)	47.03 (19.59)	64.97 (21.75)	56.77 (25.71)
Folder_4	47.35 (20.26)	39.39 (21.63)	50.61 (24.27)	47.35 (20.26)	66.52 (18.68)	56.94 (22.28)
**Hole-puncher**	**41.07 (15.65)**	**36.02 (18.07)**	**42.33 (20.12)**	**41.07 (15.65)**	**57.98 (14.86)**	**56.62 (21.62)**
Hole-puncher_1	40 (19.91)	34.45 (19.74)	39.45 (22.21)	40 (19.91)	57.87 (18.97)	56.35 (23.96)
Hole-puncher_2	41.94 (21.03)	38.74 (20.62)	43.06 (23.58)	41.94 (21.03)	54.97 (18.45)	58.74 (25.30)
Hole_puncher_3	43.16 (17.45)	36.55 (20.14)	44.74 (22.61)	43.16 (17.45)	60.16 (18.63)	53.68 (22.08)
Hole_puncher_4	39.19 (21.12)	34.35 (18.54)	42.06 (20.83)	39.19 (21.12)	58.94 (16.20)	57.71 (23.01)
**Stapler**	**46.26 (15.93)**	**37.47 (17.89)**	**44.23 (20.05)**	**46.26 (15.93)**	**46.6 (13.54)**	**55.24 (22.20)**
Stapler_1	49.23 (18.36)	38.77 (20.08)	45.74 (21.65)	49.23 (18.36)	46.48 (15.90)	57.55 (24.26)
Stapler_2	46 (16.12)	37.58 (17.93)	44.19 (21.37)	46 (16.12)	47.42 (15.29)	55.23 (23.66)
Stapler_3	45.55 (22.39)	35.48 (21.22)	43.23 (23.04)	45.55 (22.39)	45.84 (15.70)	51.45 (24.48)
Stapler_4	44.26 (18.58)	38.03 (19.55)	43.74 (23.31)	44.26 (18.58)	46.68 (19.01)	56.74 (22.16)

Note. Ratings on fotorealistic stimuli were reported on a visual analogue scale ranging from 0 to 100. 100 is reflecting a high score on the corresponding scale, whereas 0 is reflecting a low score. Mean ratings and standard deviations per category and per item are reported
