# Supplementary material for: Behavioural biases in the interaction with food objects in virtual reality and its clinical implication for binge eating disorder
Source: Eat Weight Disord. 2023 May 24;28(1):46. doi: 10.1007/s40519-023-01571-2 (PMC10209312; doi:10.1007/s40519-023-01571-2)
Supplement: Supplementary file 3 — Supplementary file3 (PDF 15 KB) [file 40519_2023_1571_MOESM3_ESM.pdf]

Online Resource 3

(Behavioural biases in the interaction with food objects in virtual reality and its clinical implication for binge eating disorder; Eating and Weight Disorders – Studies on Anorexia, Bulimia and Obesity; Max, Schag, Giel, Plewnia; University Hospital Tübingen, Tübingen Center for Mental Health, Department of Psychiatry and Psychotherapy, Neurophysiology & Interventional Neuropsychiatry, Calwerstraße 14, 72076 Tübingen – Germany, christian.plewnia@med.uni.tuebingen.de)

Reaction times without standardization

	**Mean RT Balls (SD)**				**Mean RT Food (SD)**				**Mean RT Office tools (SD)**			
	**T0**		**T1**		**T0**		**T1**		**T0**		**T1**	
	Sham	Verum	Sham	Verum	Sham	Verum	Sham	Verum	Sham	Verum	Sham	Verum
**Movement onset**	613.36 (115.39)	605.16 (97.77)	550.72 (77.94)	591.53 (79.22)	656.80 (115.93)	664.51 (119.12)	610.18 (88.54)	640.51 (88.79)	681.07 (113.63)	703.37 (86.13)	627.28 (113.42)	673.41 (88.68)
**Collection time**	1057.96 (284.00)	863.00 (221.51)	914.82 (207.41)	815.71 (180.04)	1091.92 (283.81)	1010.74 (260.99)	1003.99 (223.28)	849.82 (188.98)	1104.93 (276.89)	890.56 (241.75)	929.18 (209.46)	828.20 (185.23)

Note. The table depicts raw mean reaction times and standard deviations of the movement onset and collection time for each stimulus category (Balls, Food, Office tools) dependent on the measurement time (T0, T1) and stimulation (sham, verum).
