# Supplementary material for: Behavioural biases in the interaction with food objects in virtual reality and its clinical implication for binge eating disorder
Source: Eat Weight Disord. 2023 May 24;28(1):46. doi: 10.1007/s40519-023-01571-2 (PMC10209312; doi:10.1007/s40519-023-01571-2)
Supplement: Supplementary file 4 — Supplementary file4 (PDF 101 KB) [file 40519_2023_1571_MOESM4_ESM.pdf]

Online Resource 4

(Behavioural biases in the interaction with food objects in virtual reality and its clinical implication for binge eating disorder; Eating and Weight Disorders – Studies on Anorexia, Bulimia and Obesity; Max, Schag, Giel, Plewnia; University Hospital Tübingen, Tübingen Center for Mental Health, Department of Psychiatry and Psychotherapy, Neurophysiology & Interventional Neuropsychiatry, Calwerstraße 14, 72076 Tübingen – Germany, christian.plewnia@med.uni.tuebingen.de)

Descriptive statistics for the sample (N = 31)

**Scale (Ranges)**	**T0 Mean(*SD; Ranges*)**	**T1 Mean(*SD; Ranges*)**
Education	*N* = 20 High-School	*N* = 20 High-School
Age	36.26(13.37)	
Sex	*N* = 24 women	
FCQ total score (15-75)	38.26(13.45; 15-65)	37.97(13.05; 15-72)
FCQ desire to eat/loss of control (6-30)	15.03(5.97; 6-30)	15.16(5.66; 6-27)
FCQ reinforcement/positive affect (6-30)	14.97(5.81; 6-27)	14.58(5.93; 6-30)
FCQ hunger (3-15)	8.26(3.09; 3-13)	8.23(3.16; 3-15)
BIS total score (15-60)	31(6.90; 16-42)	33.10(6.80; 20-45)
BIS non-planning impulsivity (5-20)	10.65(3.27; 5-18)	11.45(4.07; 5-20)
BIS motor impulsivity (5-20)	10.55(2.81; 6-15)	10.06(3.15; 5-15)
BIS attentional impulsivity (5-20)	9.81(2.95; 5-20)	11.58(4.22; 5-20)
EDE total score (0-6)	2.61(0.89; 0.82-4.61)	2.04(0.85; 0.4-3.66)
EDE restraint (0-6)	1.92(1.09; 0-3.8)	1.51(1.17; 0-4.8)
EDE eating concern (0-6)	1.49(1.12; 0-4.8)	0.91(0.76; 0-3.2)
EDE weight concern (0-6)	3.36(1.29; 0.2-5.6)	2.81(1.26; 0-4.6)
EDE shape concern (0-6)	3.67(1.35; 0.13-6)	2.93(1.30; 0-5)
EDE number of binge eating episodes during the last 4 weeks	17.35(10.74; 1-40)	6.26(6.66)
TFEQ restraint (0-21)	8.42(4.51; 3-18)	8.81(5.13; 1-19)
TFEQ disinhibition (0-16)	12.39(2.32; 6-15)	10.32(3.22; 4-15)
TFEQ hunger (0-14)	9.90(2.99; 3-14)	8.03(3.70; 0-14)
UPPS urgency (1-4)	2.61(0.52; 1.42-3.42)	2.51(0.50; 1.33-3.33)
UPPS lack of premeditation (1-4)	2.11(0.53; 1-3.27)	2.08(0.46; 1.27-3.18)
UPPS lack of perseverance (1-4)	2.05(0.51; 1.1-3.1)	2.05(0.43; 1.2-2.9)
UPPS sensation seeking (1-4)	2.56(0.63; 1.33-3.83)	2.58(0.63; 1.33-3.83)
BMI	35.02(9.72)	34.90(9.62)
