# Supplementary material for: Behavioural biases in the interaction with food objects in virtual reality and its clinical implication for binge eating disorder
Source: Eat Weight Disord. 2023 May 24;28(1):46. doi: 10.1007/s40519-023-01571-2 (PMC10209312; doi:10.1007/s40519-023-01571-2)
Supplement: Supplementary file 5 — Supplementary file5 (PDF 102 KB) [file 40519_2023_1571_MOESM5_ESM.pdf]

Online Resource 5

(Behavioural biases in the interaction with food objects in virtual reality and its clinical implication for binge eating disorder; Eating and Weight Disorders – Studies on Anorexia, Bulimia and Obesity; Max, Schag, Giel, Plewnia; University Hospital Tübingen, Tübingen Center for Mental Health, Department of Psychiatry and Psychotherapy, Neurophysiology & Interventional Neuropsychiatry, Calwerstraße 14, 72076 Tübingen – Germany, christian.plewnia@med.uni.tuebingen.de)

Description of stimuli

**Food**	**Office**	**Balls**
Cheeseburger in a white wheat roll	Blue hole-puncher	New white baseball
Cheeseburger in a dark wheat roll	Pink hole-puncher	New yellow baseball
Double cheeseburger in a white wheat roll	Red hole-puncher	Used white baseball
Double cheeseburger in a dark wheat roll	Gold hole-puncher	Used yellow baseball
Chocolate cupcake with white icing and cherry	White folder	Blue hand ball
Chocolate cupcake with pink icing	Brown folder	Brown hand ball
Vanilla cupcake with white icing and cherry	Pink folder	Pink hand ball
Vanilla cupcake with chocolate icing	Red folder	Red hand ball
Donut with chocolate icing	Blue stapler	Blue-white-red beach ball
Donut with pink icing	Pink stapler	Purple-white-pink beach ball
Donut with chocolate icing and sprinkles	Red stapler	Yellow-white-brown beach ball
Donut with pink icing and sprinkles	Gold stapler	Green-white-gold beach ball
Pizza with olives, mozzarella and cheese	Brown calculator	Green tennis ball
Pizza with salami and cheese	Pink calculator	Yellow tennis ball
Pizza margherita	Red calculator	Yellow-red tennis ball
Pizza with mozzarella and basil	Gold Calculator	Pink tennis ball
